# Identification and Evaluation of the Face System of a Child Android Robot Affetto for Surface Motion Design

**DOI:** 10.3389/frobt.2018.00119

**Published:** 2018-10-23

**Authors:** Hisashi Ishihara, Binyi Wu, Minoru Asada

**Affiliations:** ^1^Adaptive Machine Systems, Graduate School of Engineering, Osaka University, Suita, Japan; ^2^SAKIGAKE/PRESTO, Japan Science and Technology Agency (JST), Tokyo, Japan

**Keywords:** android robot, face, system identification, motion capture, surface deformation

## Abstract

Faces of android robots are one of the most important interfaces to communicate with humans quickly and effectively, as they need to match the expressive capabilities of the human face, it is no wonder that they are complex mechanical systems containing inevitable non-linear and hysteresis elements derived from their non-rigid components. Identifying the input-output response properties of this complex system is necessary to design surface deformations accurately and precisely. However, to date, android faces have been used without careful system identification and thus remain black boxes. In this study, the static responses of three-dimensional displacements were investigated for 116 facial surface points against a discrete trapezoidal input provided to each actuator in the face of a child-type android robot Affetto. The results show that the response curves can be modeled with hysteretical sigmoid functions, and that the response properties of the face actuators, including sensitivity, hysteresis, and dyssynchrony, were quite different. The paper further proposes a design methodology for surface motion patterns based on the obtained response models. Design results thus obtained indicate that the proposed response properties enable us to predict the design results, and that the proposed design methodology can cancel the differences among the response curves of the actuators. The proposed identification and quantitative evaluation method can be applied to advanced android face studies instead of conventional qualitative evaluation methodologies.

## 1. Introduction

Robot faces are important information display devices that show several types of communication cues such as intention, attention, emotion, and demand, with the combined deformations of several facial parts. Movable mechanical parts of the android robot's face are covered with a flexible skin-like sheet to exhibit spatially-continuous lifelike surface deformations (Kobayashi et al., 1994, 2000, 2003; Weiguo et al., 2004; Hanson et al., 2005; Berns and Hirth, 2006; Hashimoto et al., 2006, 2008; Oh et al., 2006; Lee et al., 2008; Allison et al., 2009; Becker-Asano et al., 2010; Becker-Asano and Ishiguro, 2011; Tadesse and Priya, 2012; Cheng et al., 2013; Chihara et al., 2013; Yu et al., 2014; Asheber et al., 2016; Glas et al., 2016; Lin et al., 2016). The skin sheet is supported by skull-shaped shells to maintain a life-like shape and is connected to internal movable mechanical parts at several points. These movable parts are driven by an actuation system that moves the connection points, and their displacements are propagated on the skin around them according to both the stiffness distributions of the skin sheet and the friction conditions between the skin sheet and internal shells. Thus, an android robot face can be regarded as a system in which the inputs are actuation commands, and the outputs are surface displacement distributions.

Controlling the surface displacements of each point on the face is crucially important but difficult in android robotics because the face system contains non-linear and hysteresis elements, which are difficult to identify and tune in the design stage. For example, the distribution of surface displacements depends largely on the spatially-uneven curvature, thickness, and elasticity of the skin sheet, which often vary in skin fabrication processes. Moreover, the static and dynamic frictions between the skin sheet and shells cause motion hysteresis, and the frictions may change temporally depending on subtle fluctuations in the contact conditions between the skin sheet and shells.

The input-output properties of this complex non-linear system must be identified in order to design the output (or surface displacement distribution) precisely and investigate problematic system components for future improvement of the face system. However, android faces have been used without identifying their system properties carefully. Namely, they have been used as black boxes. Although the depth changes of facial surfaces and the displacements of several facial surface points have been measured in several android robot studies (Hashimoto et al., 2008; Tadesse and Priya, 2012; Cheng et al., 2013), motion control was not the focus of these studies, perhaps because facial expressions of representative categorical affects such as happiness and anger, can be realized to a certain extent through manual input tuning. However, this trial-and-error tuning is not sufficient for advanced quantitative studies, such as, for example, designing surface motions from one deformation state to another, and quantitatively evaluating the subtle differences of facial deformation between robots and humans.

**Figure 1:**
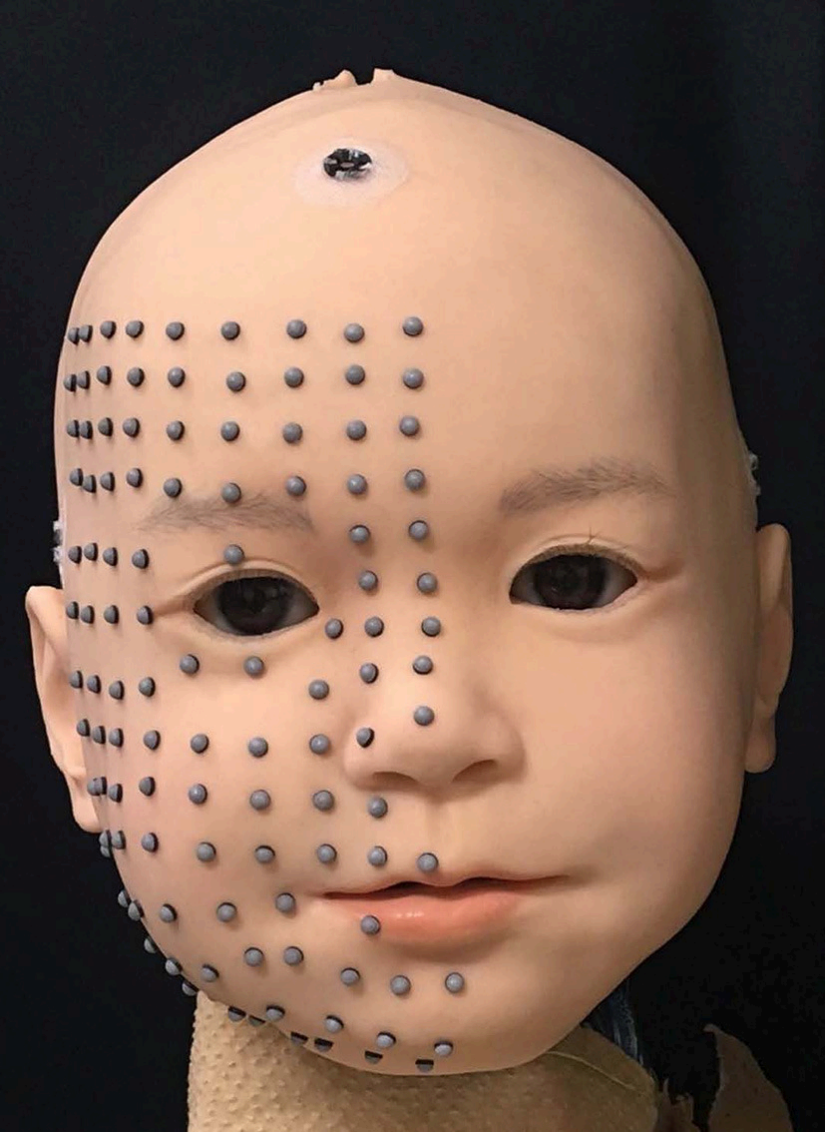
Facial appearance of Affetto (model 2018), with hair detached for the study.

In this study, we investigated the static responses of three-dimensional displacements of facial surface points of the child-type android robot Affetto (model 2018) with a discrete trapezoidal input to each facial actuator. Totally 116 surface points were selected in the right half of the face, as shown in Figure 1. This face is an upgraded version of one (model 2011) introduced in previous studies (Ishihara et al., 2011; Ishihara and Asada, 2015). The upgraded version has more actuators (for a total of 22 DoFs), a realistic appearance, and a more reliable actuation mechanism than the first one. The system identification experiment presented here used 16 DoFs to deform the skin sheet.

Based on the displacements measured with a motion capture system, the spatial distributions and the linearities of the spatial transitions of displacements were investigated for each surface point against each deformation unit (DU), defined as a set of facial deformation mechanisms including one actuator and the transmissions required to realize a distinctive facial skin surface deformation pattern. The response curves of the displacements were modeled against input command sequences with sigmoid functions for each DU to represent non-linear static response properties, such as sensitivity and hysteresis. In addition, the dyssynchronies of the response curves among all surface points were also evaluated because the response curves may vary among surface points. Design methodologies for surface motion patterns were proposed based on the obtained response models for each DU, and experiments were conducted for several motion patterns to validate the effectiveness of the proposed design methodology and evaluation properties.

## 2. Methods

### 2.1. Robot

**Figure 2:**
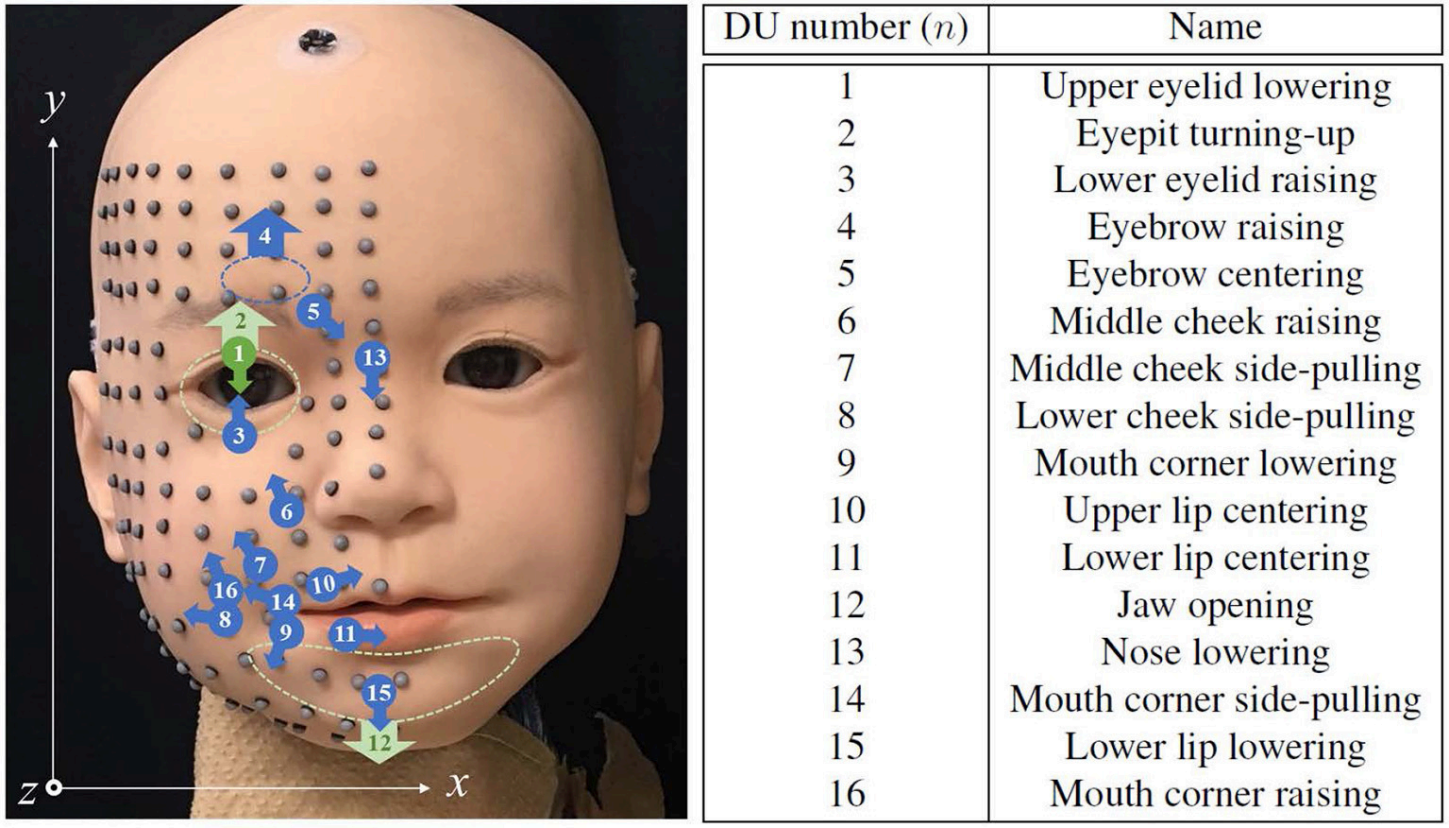
Approximate connecting point positions and representative displacement direction of each DU, the identification numbers and names of which are summarized at right. Marker locations and the coordinate system for representing their positions are also depicted.

Figure 1 shows the appearance of the newly-developed face used in this study, developed in collaboration with A-Lab Co.,Ltd. for a child android robot, Affetto (Ishihara et al., 2011; Ishihara and Asada, 2015). The face has a total of 22 DoFs. Sixteen of which were selected to implement the facial deformation for this study. All actuators for these DoFs are pneumatic actuators, including linear cylinders and rotary vane actuators. The approximate positions of connection points and representative directions of surface displacement for each DU are depicted in Figure 2. The names of the *n*-th DU (*n* = 1, ⋯ , 16) are also listed in the figure; blue indicates the DUs without sensory feedback (open-loop control), and green indicates the DUs with sensory feedback (closed-loop control); and the dotted loops indicate the approximate regions where the skin sheet and movable mechanical parts were connected for each DU. The positions and directions of each DU represented in Figure 2 do not correspond exactly with the actual positions and directions of the displacements.

**Figure 3:**
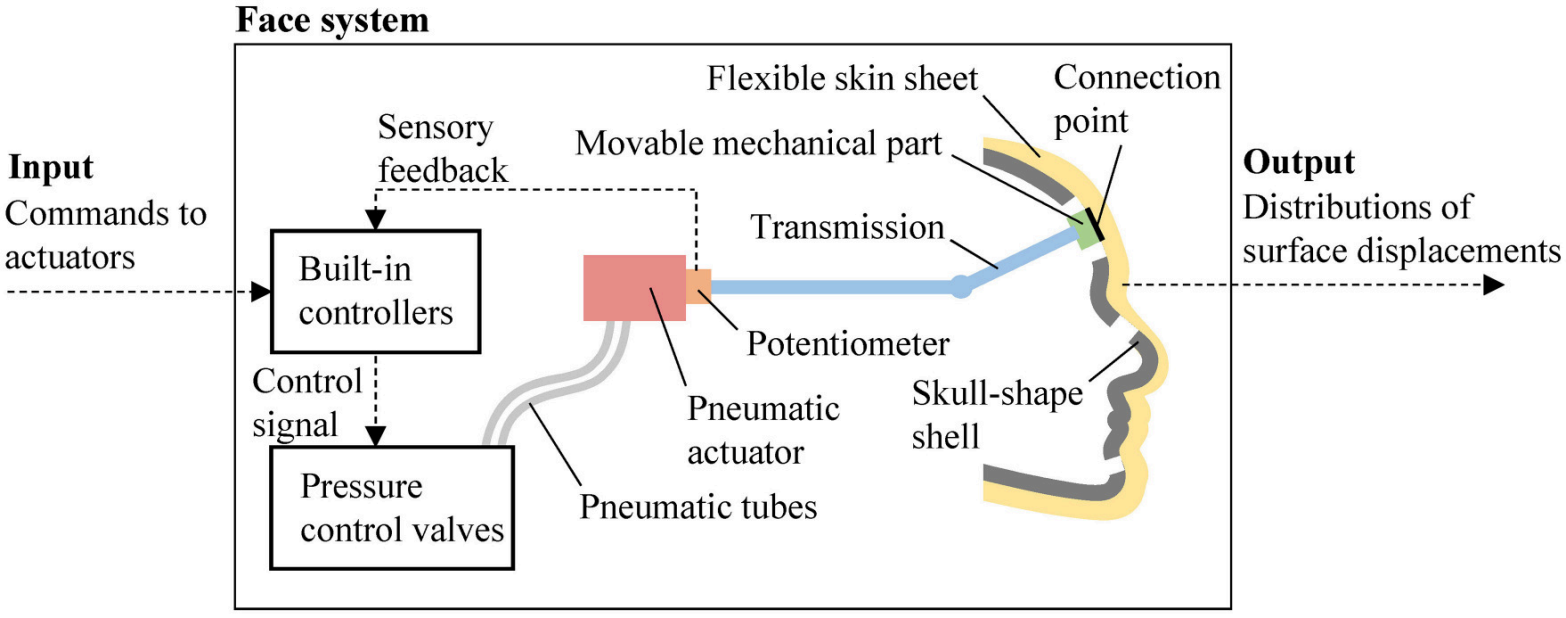
Schematic diagram of Affetto face system.

Figure 3 shows the system overview of the face of Affetto. The face system includes the skin sheet, skull-shaped shells, movable mechanical parts, transmissions, pneumatic actuators, potentiometers, built-in controllers, and pressure control valves. Some of the DUs have potentiometers to measure the length or rotation angle of their rods to realize closed-loop control, whereas the remaining DUs without potentiometers are controlled with an open loop because of the limited space. Each actuator rod is connected to a movable mechanical part attached on the internal side of the silicone skin sheet rubber via a transmission such as a tendon-based mechanism, a linkage mechanism, or a gear mechanism.

The target position commands (hereafter, commands) to each rod are sent from an external computer to the built-in controllers. The built-in controllers receive the sensory feedback from potentiometers to determine the control signals sent to the pressure control valves. These valves are connected to the actuators via pneumatic flexible tubes, and pressure-controlled air is sent from the valves to the actuators to provide power to drive the actuator rods. Although the ranges of motion of these actuators are different, the commands to each rod can be set with an integer ranging from 0 to 255.

### 2.2. Command schedule

**Figure 4:**
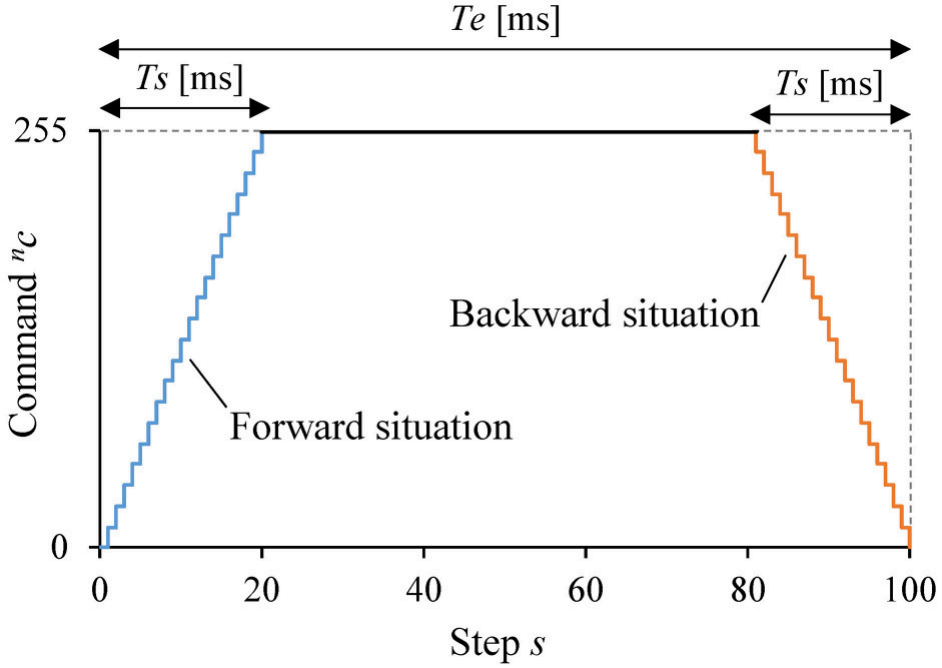
Command schedule for the static response analysis.

Figure 4 shows the 100-step discrete trapezoidal input used as a command ^*n*^*c* = 0, ⋯ , 255 to the *n*-th DU (*n* = 1, ⋯ , *N* and *N* = 16 in this study) for the static response analysis. The command was increased from 0 to 255 in 20 steps. After waiting 60 steps, the command was decreased from 255 to 0 in 20 steps. The step time was approximately 50 ms. The situation when the command is increasing is hereafter called a forward situation; when the command is decreasing is hereafter called a backward situation. The command ^*n*^*c* at step *s* in the forward situation was determined as ${}^{n}c_{|s}=\left\lfloor 255s/20\right\rfloor$ (*s* = 1, ⋯ , 20); ^*n*^*c* at step *s* in the backward situation was determined as ${}^{n}c_{|s}=\left\lfloor 255\left(100-s\right)/20\right\rfloor$ (*s* = 81, ⋯ , 100). The commands between these situations were 255. This trapezoidal input was provided to the *n*-th DU one by one, while the commands for the DUs other than the *n*-th one were 0.

### 2.3. Measurement

Facial deformations of the robot according to the trapezoidal input were measured as combinations of the three-dimensional positions of several surface points on the skin sheet. The spatial transitions of the surface points were tracked with six optical motion capture cameras (OptiTrack Flex13) at 120 frames per second. The first measurement time *t* = 0 [frame] was matched to the timing of the command step *s* = 0. For the motion capturing, a total of 116 reflective markers of 3-mm diameter were attached on the right half of the facial surface as shown in Figure 2. These markers were aligned at approximately 10-mm intervals so that several of them were located at morphological feature points, e.g., the middle point of the upper eyelid and the corner of the lip.

The locations and directions of these motion capture cameras were determined so that at least three cameras could capture each marker. The averages of ten captured positions of the *m*-th surface point (*m* = 1, ⋯ , *M* and *M* = 116 in this study) when the *n*-th DU was actuated at *t* were converted to time-sequence three-dimensional positions ${}^{n}v_{m|t}={{[}^{n}x_{m|t}{\;}^{n}y_{m|t}{\;}^{n}z_{m|t}]}^{\text{T}}$ by motion capture software (OptiTrack Motive 2.0.0) in the coordinate system shown in Figure 2. We define the trajectory of the spatial transition of the *m*-th marker produced by the *n*-th DU as ${}^{n}\Phi_{m}={[}^{n}v_{m|t=0},\cdots ,{\;}^{n}v_{m|t=T_{e}}]$, and we define the facial posture at *t*, or the state of the facial surface deformation, as Ψ_|*t*_ = [*v*_1|*t*_, ⋯ , *v*_*m*|*t*_, ⋯ , *v*_*M*|*t*_].

Measurement errors were minimized by calibration with a rod having several precisely located markers and reduced to 0.04 mm on average. In addition, oscillative measurement noise was measured with the face in an inactive state as the square root of the standard deviation for each of three orthogonal directions and found to be 0.025 mm on average for all markers. Based on these values, we disregarded the measured trajectories of which maximum displacements were smaller than 0.05 from the subsequent analyses.

Furthermore, we calculated the averaged standard deviations of the displacements over 10 measurements in the forward and backward situations for every marker. Almost all markers had small values of < 0.03 mm, whereas one marker on the nose bridge had an exceptionally large value over 0.1 mm. The large fluctuation of this marker was considered to be not an actual fluctuation but caused by a local capturing error due to its position in the shadow behind the nose. Therefore, we disregarded this marker in the subsequent analyses.

### 2.4. Analysis

This section introduces the spatial deformation and static response analysis methods applied in this study. Two deformation properties are introduced in section 2.4.1 for spatial deformation analysis, and three response properties are introduced in section 2.4.2 for static response analysis. Table 1 summarizes the symbol definitions used in these analyses.

**Table 1:** Symbol definition for the spatial deformation and static response analyses.

**Symbol**	**Symbol name**	**Type**	**Meaning**
*m*	Surface marker number	Scalar	Identification number of 116 markers
*n*	DU number	Scalar	Identification number of 16 DUs
^*n*^*c*	Command	Scalar	Integer target position command from 0 to 255 sent to *n*-th DU
*t*	Measurement time	Scalar	Elapsed data frames since the beginning of the forward situation
${}^{n}v_{m|t}$	Marker position	Vector	Three-dimensional position vector of *m*-th marker at *t* when *n*-th DU is actuated
${}^{n}\Phi_{m}$	Spatial trajectory	Matrix	Time sequence of ${}^{n}v_{m|t}$
Ψ_|*t*_	Facial posture	Matrix	Array of the marker position vector *v*_*m*_ at *t*
${}^{n}d_{m|t}$	Displacement vector	Vector	Displacement vector of *m*-th marker produced by *n*-th DU at time *t*
${}^{n}\delta_{m|t}$	Normalized displacement	Scalar	Length of the displacement vector ${}^{n}d_{m|t}$ normalized into [0, 1] based on its maximum value among every *t*
${}^{n}l_{m}$	Spatial linearity	Scalar	Evaluation index value of spatial linearity of ${}^{n}\Phi_{m}$
^*n*^*R*	Response function	Function	Function of ^*n*^*c* representing to what extent ${}^{n}\delta_{m}$ is produced by the command ^*n*^*c* at a facial surface on average
^*n*^α	Sensitivity	Scalar	Evaluation index value of to what extent a facial surface moves on average according to a subtle change of the command sent to *n*-th DU
^*n*^β	Hysteresis	Scalar	Evaluation index value of how much the response curves against *n*-th DU are different between the forward and backward situations
^*n*^γ	Dyssynchroly	Scalar	Evaluation index value of how much the response curves against *n*-th DU are different among different facial surface positions

#### 2.4.1. Spatial deformation analysis

The spatial trajectory ${}^{n}\Phi_{m}$ of the *m*-th marker produced by the *n*-th DU should be different for every *m* and *n*. Therefore, we should know the vector fields of the displacement vectors ${}^{n}d_{m|t}$ of every marker against every DU. Furthermore, we should investigate how straight the spatial trajectories are because the spatial deformation analysis becomes complicated if the trajectories deviate.

These spatial properties, namely the displacement vector ${}^{n}d_{m|t}$ and spatial linearity ${}^{n}l_{m}$ of a trajectory ${}^{n}\Phi_{m}$, were calculated as follows:

1. **Displacement vector** ${}^{n}d_{m|t}$: Calculated by subtracting the initial position vector of the *m*-th marker from its position vector at *t*: ${}^{n}d_{m|t}{=}^{n}v_{m|t}{-}^{n}v_{m|t=0}$.
2. **Spatial linearity** ${}^{n}l_{m}$: Calculated as the contribution ratio of the first principal spatial distribution component for every measured position in ${}^{n}\Phi_{m}$ over 10 measurements: ${}^{n}l_{m}=\left[0,1\right]$.

#### 2.4.2. Static response analysis

The lengths of the displacement vectors change according to the command. Let us define ${}^{n}\delta_{m}=\left[0,1\right]$ as a normalized displacement of the *m*-th marker produced by the *n*-th DU: ${}^{n}\delta_{m}={|}^{n}d_{m}|/\underset{t}{\max}{|}^{n}d_{m|t}|$. Then, we have ${}^{n}\delta_{m}={\;}^{n}R(nc)$. Here, ^*n*^*R* is a response function of the face system against the *n*-th DU. This response function represents how the command sequence is distorted by the face system: it should be a linear function if the connecting point moves precisely according to the commands and the skin surface moves smoothly according to the connecting point. However, this linearity should be distorted if the face system exhibits command-dependent deformation turbulence factors such as friction fluctuations or non-linear stiffness.

Therefore, the shape of ^*n*^*R* is the target of static response analysis in this study. Let us distinguish two types of response function by adding a circumflex ˆ and a caron ˇ because the response function might be different for the forward and backward situations; then, ${}^{n}\hat{R}$ is a forward response function and ^*n*^Ř is a backward response function. The parameters of these functions were optimized by the least squares method with the measured data obtained in the forward and backward situations, respectively. Here, the measured data were the pairs of *t* and ${}^{n}\delta_{m}$ from every *m* in each situation. First, the parameters were optimized with the pairs of *t* and ${}^{n}\delta_{m}$, and then the functions with these optimized parameters were converted to ^*n*^*R* by switching *t* with ^*n*^*c* as $t=\left\lfloor T_{s}{\;}^{n}c/255\right\rfloor$ because the time resolution of ${}^{n}\delta_{m}$ is higher against *t* than ^*n*^*c*.

We used a sigmoid function to represent ^*n*^*R*, ^*n*^*R*(^*n*^*c*) = {1−^*e*^^−α^^(^^*nc*−β)^}^−1^, because this function has two parameters, the gain parameter α and the bias parameter β, which are suitable for evaluating the two types of response properties, sensitivity and hysteresis. Sensitivity is the index used to evaluate to what extent a facial surface moves according to subtle changes in the command. Hysteresis is the index used to evaluate to what extent the response curves are different between the forward and backward situations. In addition to these evaluation indices, we propose dyssynchrony, which is the index used to evaluate to what extent the response curves are different among different surface positions.

These static response properties of an android robot, namely (1) sensitivity, (2) hysteresis, and (3) dyssynchrony against the *n*-th DU, were calculated as follows:

1. **Sensitivity** ^*n*^α: This is the average of the optimized gain parameters $\hat{\alpha }$ and $\hat{\alpha }$ of ${}^{n}\hat{R}$ and ^*n*^Ř, respectively.
2. **Hysteresis** ^*n*^β: This is calculated by subtracting the optimized bias parameters β of the backward response function from those of the forward response function: ${}^{n}\beta {=}^{n}\hat{\beta }{-}^{n}\hat{\beta }$, where ${}^{n}\hat{\beta }$ and ${}^{n}\hat{\beta }$ are β of ${}^{n}\hat{R}$ and ^*n*^Ř, respectively.
3. **Dyssynchrony** ^*n*^γ: This is calculated as the averaged standard deviations of the response curves of all markers among every measurement time. Namely, ${}^{n}\gamma =T_{e}^{-1}\Sigma_{t}\left(u_{t}\right)$, where *u*_*t*_ is the sample standard deviation of ${}^{n}\delta_{m|t}$ among every *m* at time *t*: $u_{t}={\left[{\left(M-1\right)}^{-1}\Sigma_{m}{{(}^{n}\delta_{m|t}-{\;}^{n}{\bar{\delta }}_{|t})}^{2}\right]}^{1/2}$, where ${}^{n}{\bar{\delta }}_{|t}=M^{-1}\Sigma_{m}{(}^{n}\delta_{m|t})$.

**Figure 5:**
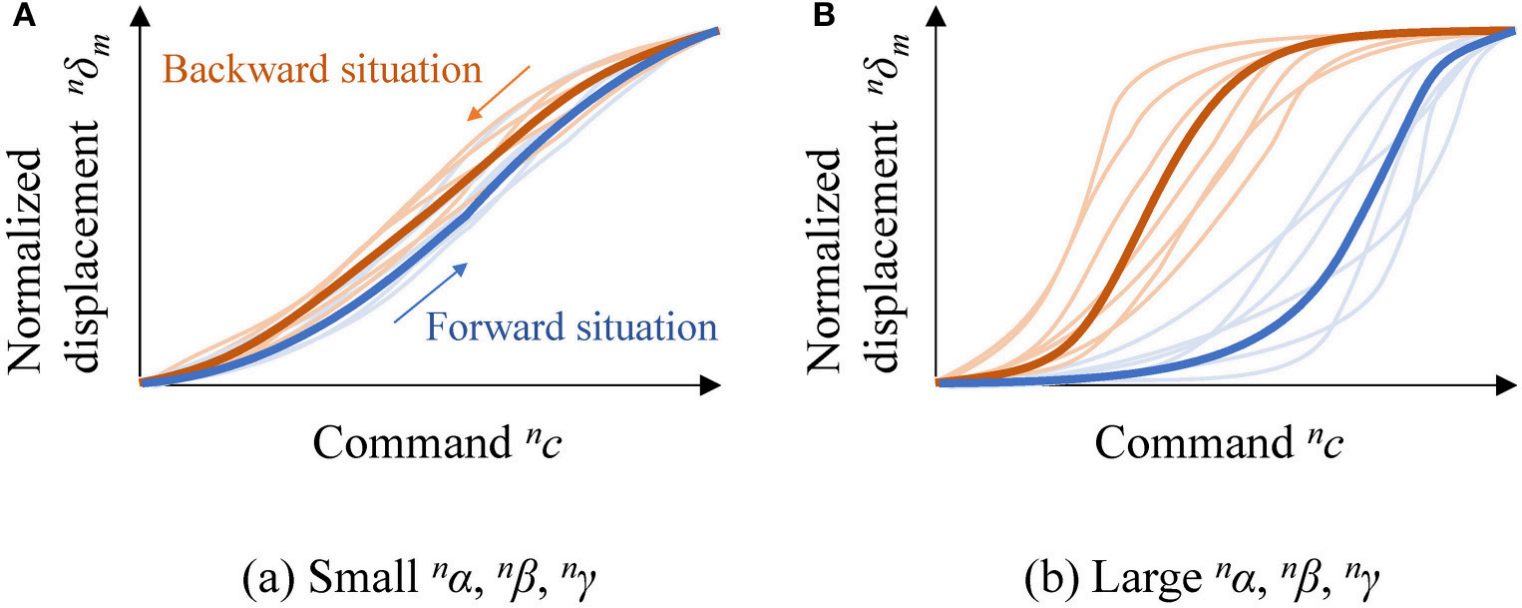
Examples of contrary response patterns: **(A)** response curves and functions against a DU with small ^*n*^α, ^*n*^β, and ^*n*^γ; and **(B)** response curves and functions against a DU with large ^*n*^α, ^*n*^β, and ^*n*^γ.

Figure 5 shows examples of two contrary response patterns. Figure 5A illustrates the response curves and functions against a DU with small ^*n*^α, ^*n*^β, and ^*n*^γ, and Figure 5B illustrates the response curves and functions against a DU with large ^*n*^α, ^*n*^β, and ^*n*^γ. In each subfigure, thin and bold curves represent response curves of several markers and two response functions, respectively; blue and red curves represent forward and backward situations, respectively. The gradients of the functions, differences between the response functions, and variations of response curves are smaller in Figure 5A than in Figure 5B because the three indices ^*n*^α, ^*n*^β, and ^*n*^γ are smaller for the case shown in Figure 5A.

The DUs with smaller ^*n*^α, ^*n*^β, and ^*n*^γ are more desirable for designing motion patterns because (1) subtle displacement control is easy to realize if the response functions have small gradients, (2) the dead time is shorter if the response functions for the forward and backward situations are similar, and (3) deformations can be represented with simple mathematical models if the response curves for all surface positions are similar.

### 2.5. Motion design

#### 2.5.1. Methodology

**Figure 6:**
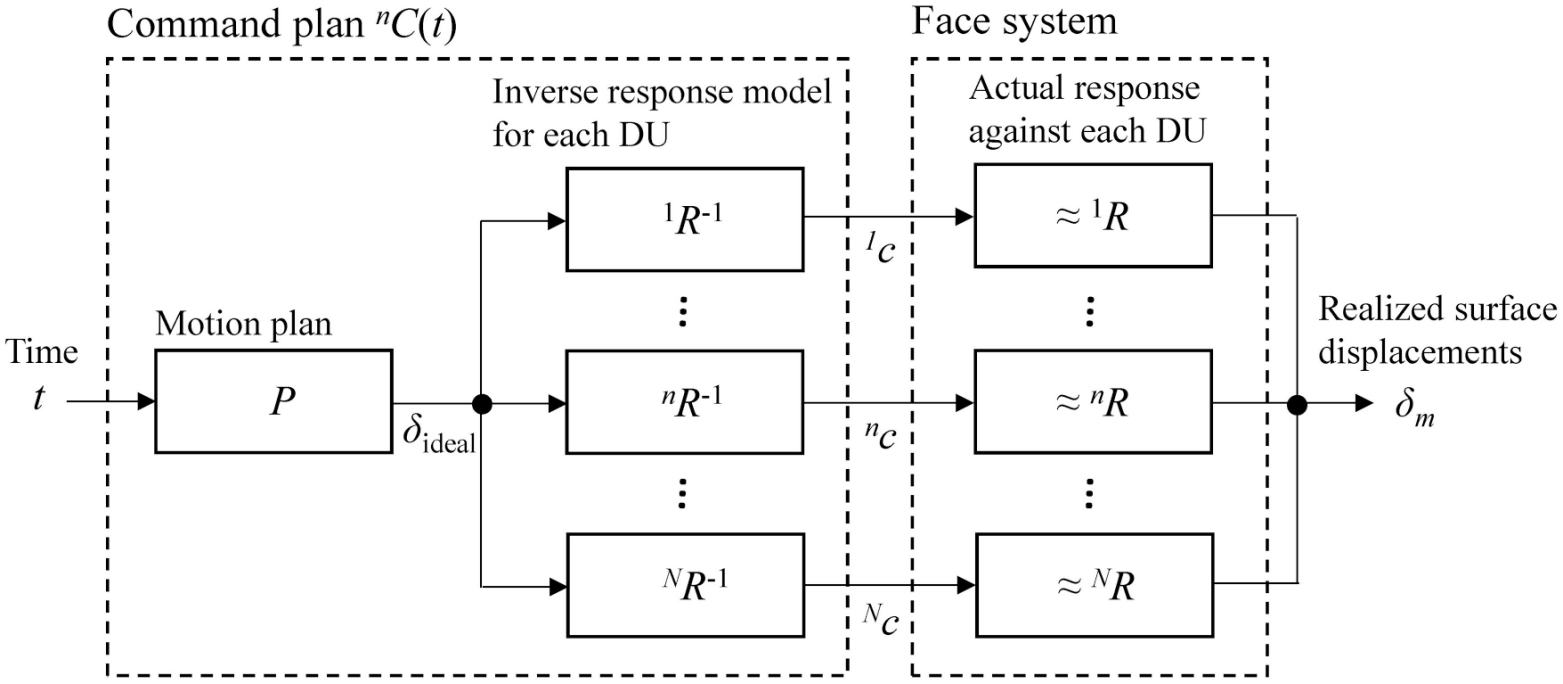
System diagram of motion design for a face system with *N* DUs.

We define motion design as the process of determining appropriate command time-sequences for several DUs so that an ideal transition of the displacements (hereinafter called an ideal motion pattern) can be realized all over the facial surface. Figure 6 shows the system diagram of motion design for a face system with *N* DUs. The face system has its own static input-output property, which can be identified as a set of response functions ^*n*^*R* for each DU. Designers should determine an appropriate command plan function ^*n*^*c* =^*n*^*C*(*t*) for each DU to realize an ideal motion pattern according to a motion plan δ_ideal_ = *P*(*t*) using several DUs. Here, δ_ideal_ is an ideal normalized displacement at *t* for all markers, while *P* is a motion plan function describing the ideal motion pattern.

In this case, designers can utilize the inverse response functions to gain the appropriate command plans ^*n*^*C*(*t*). Namely, we have ^*n*^*c* =^*n*^*C*(*t*) =^*n*^*R*^−1^(*P*(*t*)), where ^*n*^*R*^−1^ is the inverse function of ^*n*^*R*. For reference, ^*n*^*R*^−1^(δ) =^*n*^β−^*n*^α^−1^ln{(1−δ)/δ}.

#### 2.5.2. Performance evaluation of motion design

The focus of this study in the extent to which the ideal motion patterns can be realized with the proposed motion design methodology and how well the response properties correspond design results. For these evaluations, we consider three design situations to execute five motion patterns. The first situation is where one DU with large ^*n*^α (sensitivity) and ^*n*^β (hysteresis), and small ^*n*^γ (dyssynchrony) is used. The second situation is where another contrary DU with small ^*n*^α and ^*n*^β and large ^*n*^γ is used. The third situation is where DUs 1 (upper eyelid lowering), 7 (middle cheek side-pulling), and 12 (jaw opening) are used simultaneously to realize transitions between a neutral face Ψ_1_ and a smiling face Ψ_2_.

**Figure 7:**
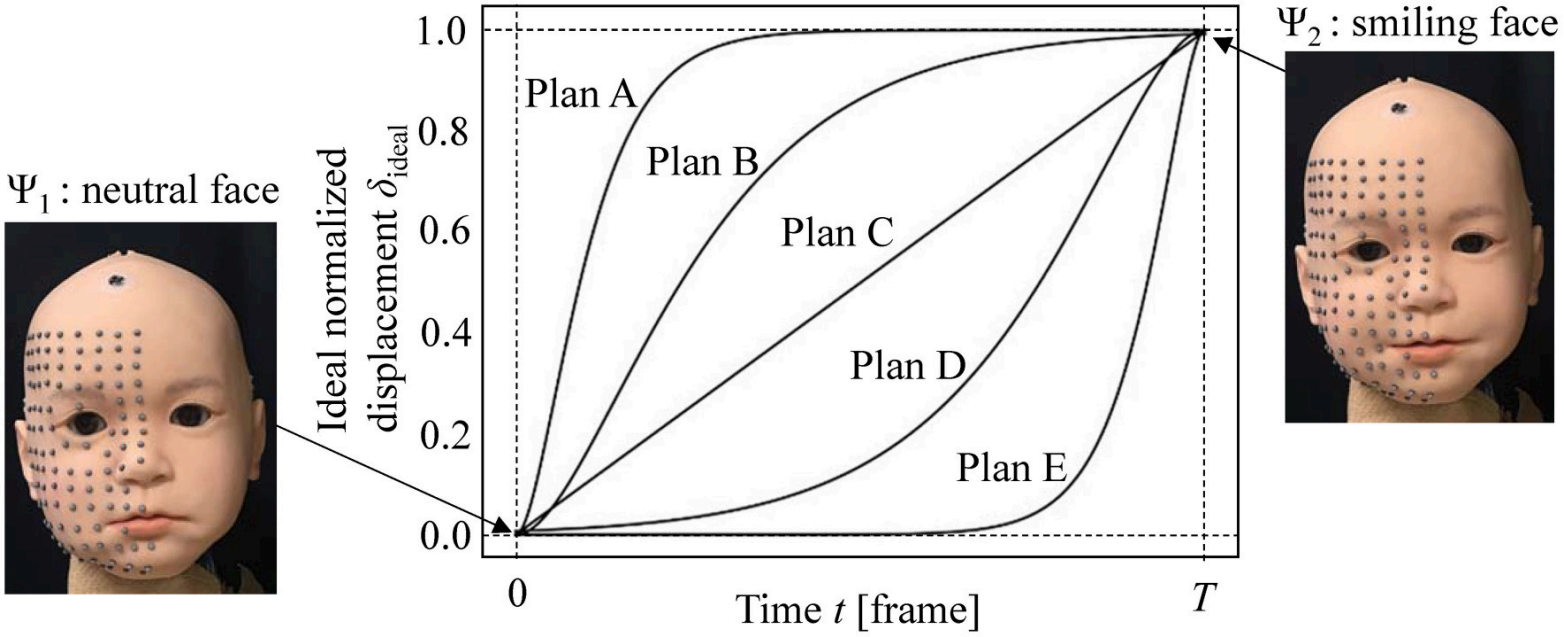
Motion plans for motion design experiment from a neutral face Ψ_1_ to smiling face Ψ_2_.

Figure 7 introduces the five motion plans A, B, C, D, and E from a neutral face Ψ_1_ to a smiling face Ψ_2_. Plan A is the most rapid one, Plan C is a linear one, and Plan E is the slowest one. Plans B and D follow a middle path between Plans A and C, and Plans C and E, respectively. The functions representing these plans were *P*(*t*) = 1−*e*^−18*t*/*T*^(1+18*t*/*T*) for Plan A; *P*(*t*) = 1−*e*^−7*t*/*T*^(1+7*t*/*T*) for Plan B; *P*(*t*) = *T*^−1^*t* for Plan C; *P*(*t*) = *e*^−7(*T*−*t*)/*T*^{1+7(*T*−*t*)/*T*} for Plan D; and *P*(*t*) = *e*^−18(*T*−*t*)/*T*^{1+18(*T*−*t*)/*T*} for Plan E. Here, *T* represents the total data frames for the motion design. Each of these plans was converted to a command plan for each DoF, and the motion patterns thus realized were measured to compare the difference between the ideal and realized motion patterns for each motion plan. In this experiment, commands were sent to DUs in 130 steps with intervals of approximately 16 ms so that the total time was approximately 2 s.

## 3. Results

### 3.1. Spatial deformation properties

**Figure 8:**
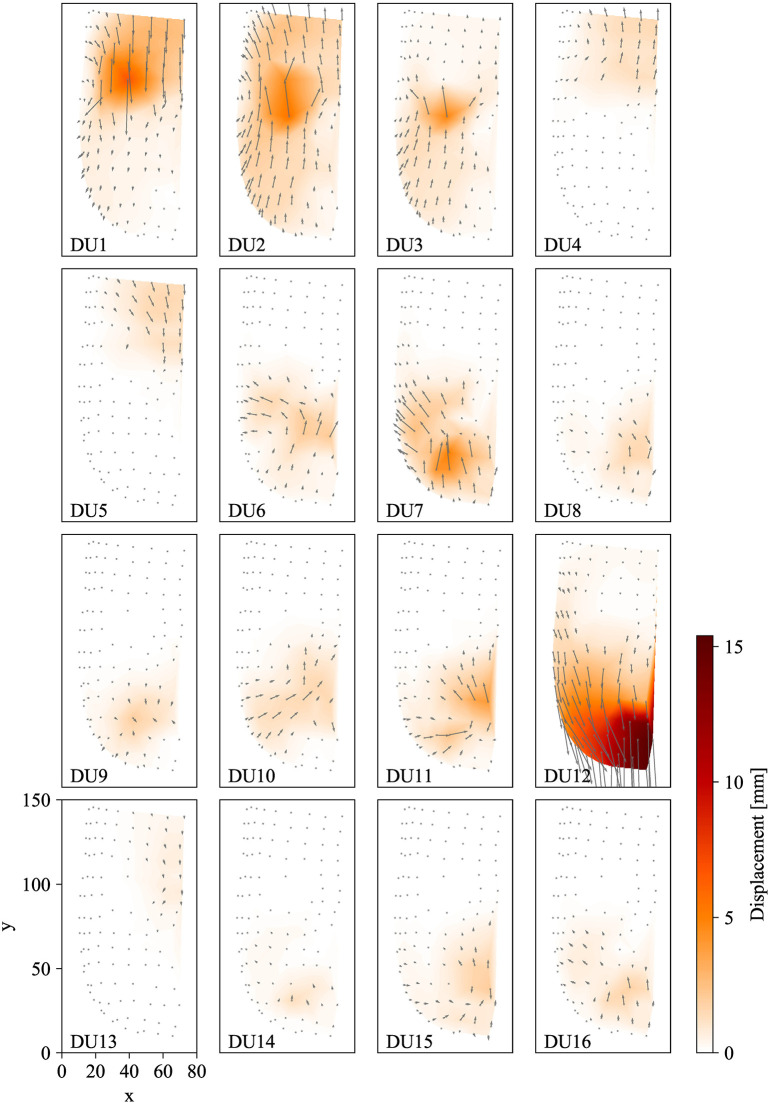
Measured displacement vector field for each DU.

Figure 8 shows the distributions of the measured displacement vectors for 16 DUs projected on the *xy*-plane, as well as heat maps of the vector length. The arrows represent the displacement vectors at ^*n*^*c* = 255. The dark red regions indicate the regions with longer displacement vectors. The arrow lengths were scaled five times larger so that the orientations of the vector could be visible.

The figure illustrates how the surface deformations differ among the DUs. For all DUs, the longest displacement vectors were observed around the connecting points. For example, displacement vectors were longest around the eye for DUs 1 (upper eyelid lowering), 2 (eyeballs turning-up), and 3 (lower eyelid raising), and their peak positions were slightly different: at the upper side, middle, and lower side of the eye, respectively.

In addition, the figure shows how widely the displacements were propagated on the facial surface. Although the vector lengths decreased with increasing distance from their peak positions, a number of markers distant from their peak positions moved more than approximately 1 mm. For example, markers were moved over 1 mm by DU 2 (eyeballs turning-up), although this DU was simply for turning the eyepits and eyeballs up and down. This wide propagation supports the necessity of evaluating dyssynchrony, as in this study.

**Figure 9:**
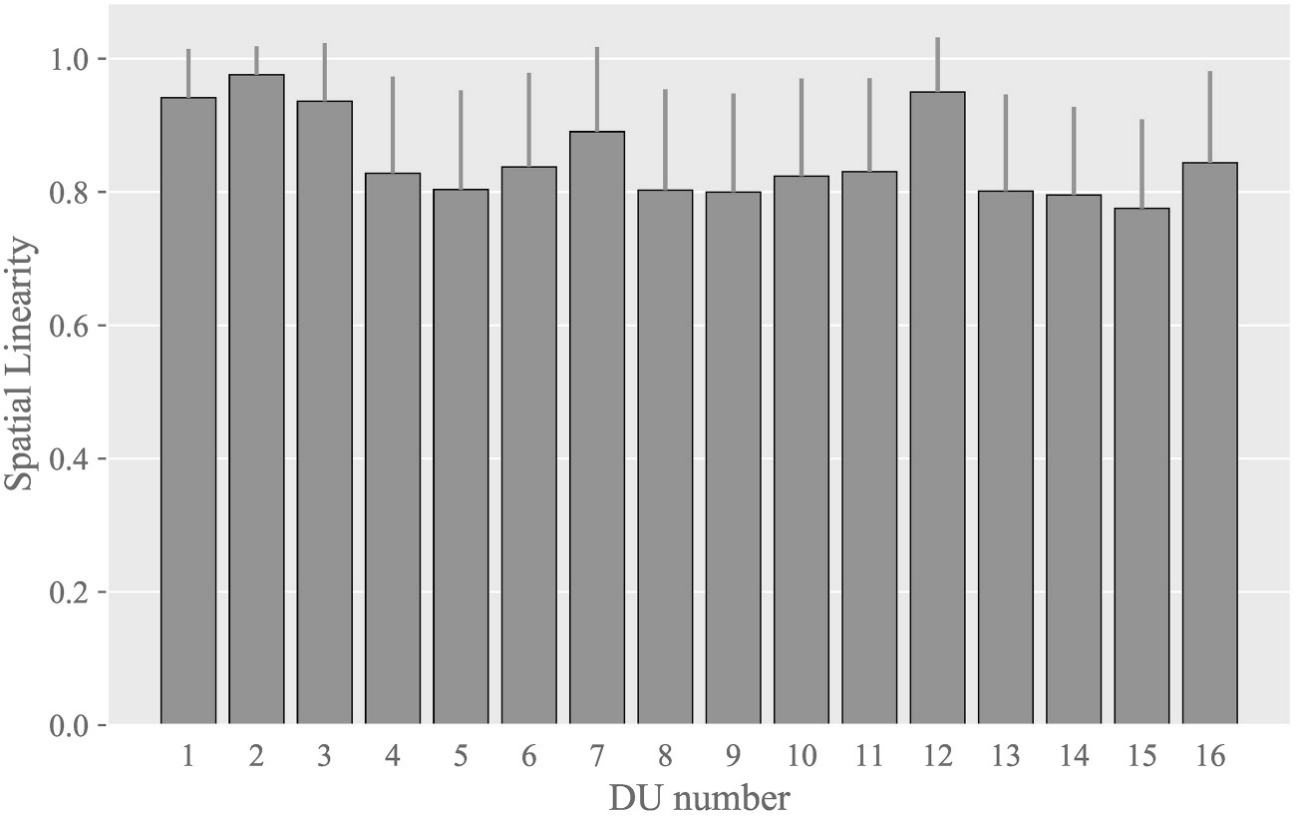
Averaged values of the spatial linearity index among all markers for each DU.

Figure 9 shows the averaged values of spatial linearity ${}^{n}l_{m}$ among all markers for each DU; the error bars indicate the sample standard deviations. The linearity was over approximately 0.9 for DUs associated with the eye (DUs 1, 2, and 3) and the jaw (DU 12), and the linearity was approximately 0.8 for the remaining DUs. These high spatial linearities indicate that the spatial trajectories of the facial markers were almost straight against all DUs. This allows us to evaluate the motion pattern of a marker along its spatial trajectory as the displacement from its initial position because these two values match each other when the trajectory is straight.

### 3.2. Static response properties

#### 3.2.1. Response curves

**Figure 10:**
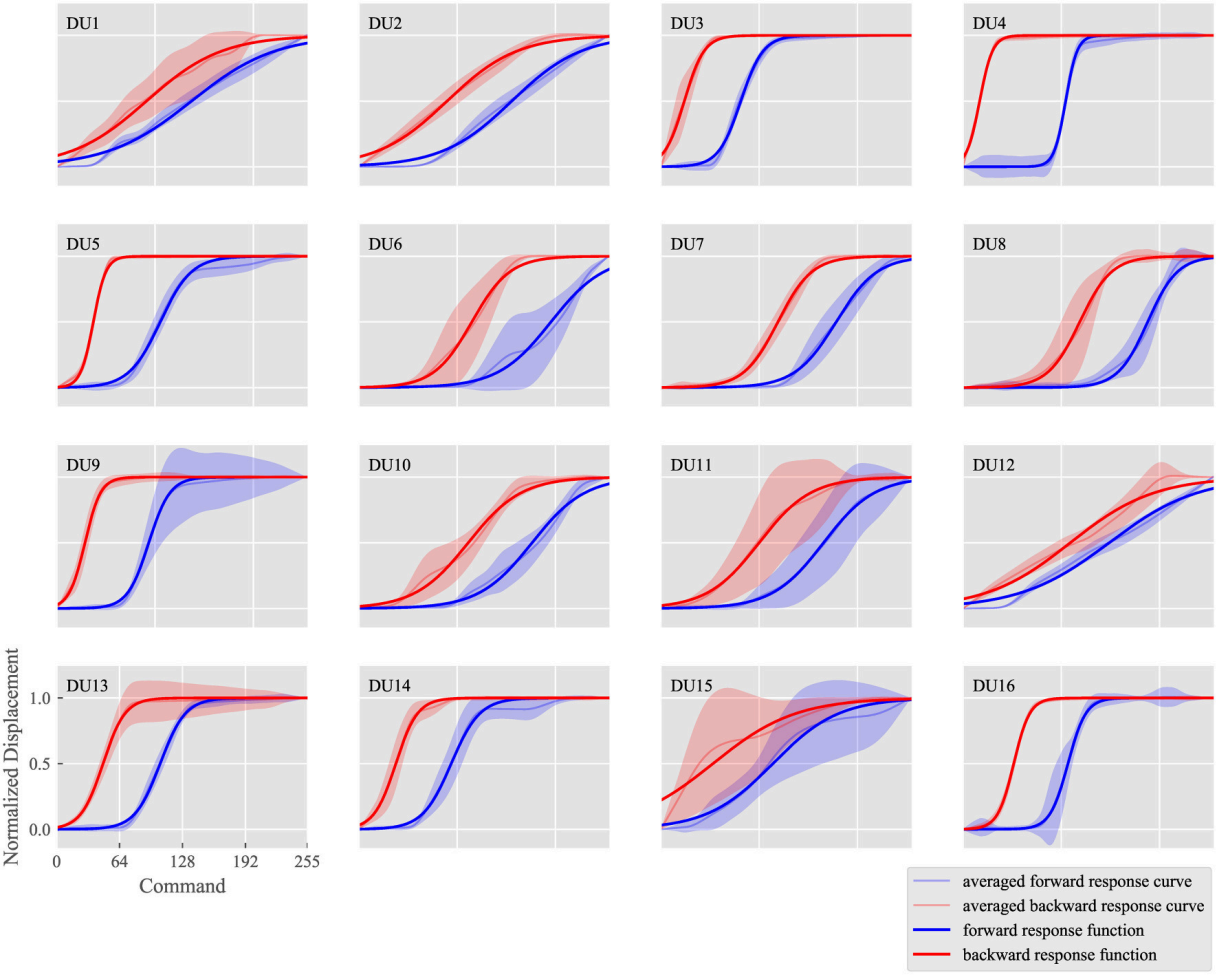
Measured response curves of ${}^{n}\delta_{m}$ and identified response functions ${}^{n}\hat{R}$ and ^*n*^Ř for each DU.

Figure 10 summarizes the measured response curves for each DU, which demonstrate how well the response curves can be modeled with sigmoid functions. The horizontal axis indicates the command ^*n*^*c* while the vertical axis indicates the normalized displacement ${}^{n}\delta_{m}$. In the subfigures for each DU, the measured response curves in the forward and backward situations are depicted by pale blue and red curves, respectively, with standard deviations of the response curves for all markers; deep blue and red curves represent the identified forward and backward response functions ${}^{n}\hat{R}$ and ^*n*^Ř, respectively.

The subfigures can be compared to analyze how different the response curves are among DUs. Response curves for DUs 4 (eyebrow raising), 5 (eyebrow centering), and 16 (mouth corner raising) show large gradients, whereas those of DUs 1 (upper eyelid lowering), 2 (eyepit turning-up), and 12 (jaw opening) show small gradients. In addition, the response functions of both situations were similar in DUs 1 (upper eyelid lowering) and 12 (jaw opening), whereas they were relatively different in DUs 4 (eyebrow raising) and 6 (middle cheek raising). Thus, each DU was considered to exhibit different sensitivity and hysteresis values. Furthermore, the standard deviations were large in DUs 11 (lower lip centering) and 15 (lower lip lowering), whereas they were small in DUs 2 (eyepit turning-up), 3 (lower eyelid raising), 4 (eyebrow raising), 5 (eyebrow centering), 7 (middle cheek side-pulling), and 16 (mouth corner raising), indicating different dyssynchrony values.

#### 3.2.2. Static response property chart

**Figure 11:**
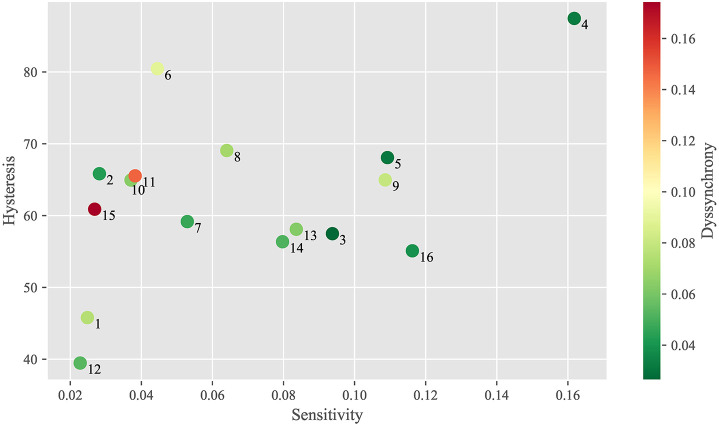
Static response property chart for each DU.

Figure 11 indicates the index values of the three static response properties for each DU. The horizontal axis indicates sensitivity ^*n*^α, while the vertical axis indicates hysteresis ^*n*^β. The marker color indicates dyssynchrony ^*n*^γ.

This chart show to what extent these properties differ among DUs. DUs 1 (upper eyelid lowering) and 12 (jaw opening) have small ^*n*^α (sensitivity) and ^*n*^β (hysteresis), whereas DU 4 (eyebrow raising) has large ^*n*^α and ^*n*^β. In addition, DUs 2 (eyepit turning-up), 11 (lower lip centering) and 15 (lower lip lowering) have similar ^*n*^α and ^*n*^β, whereas DUs 11 and 15 have larger ^*n*^γ (dyssynchrony) than DU 2.

### 3.3. Motion design

**Figure 12:**
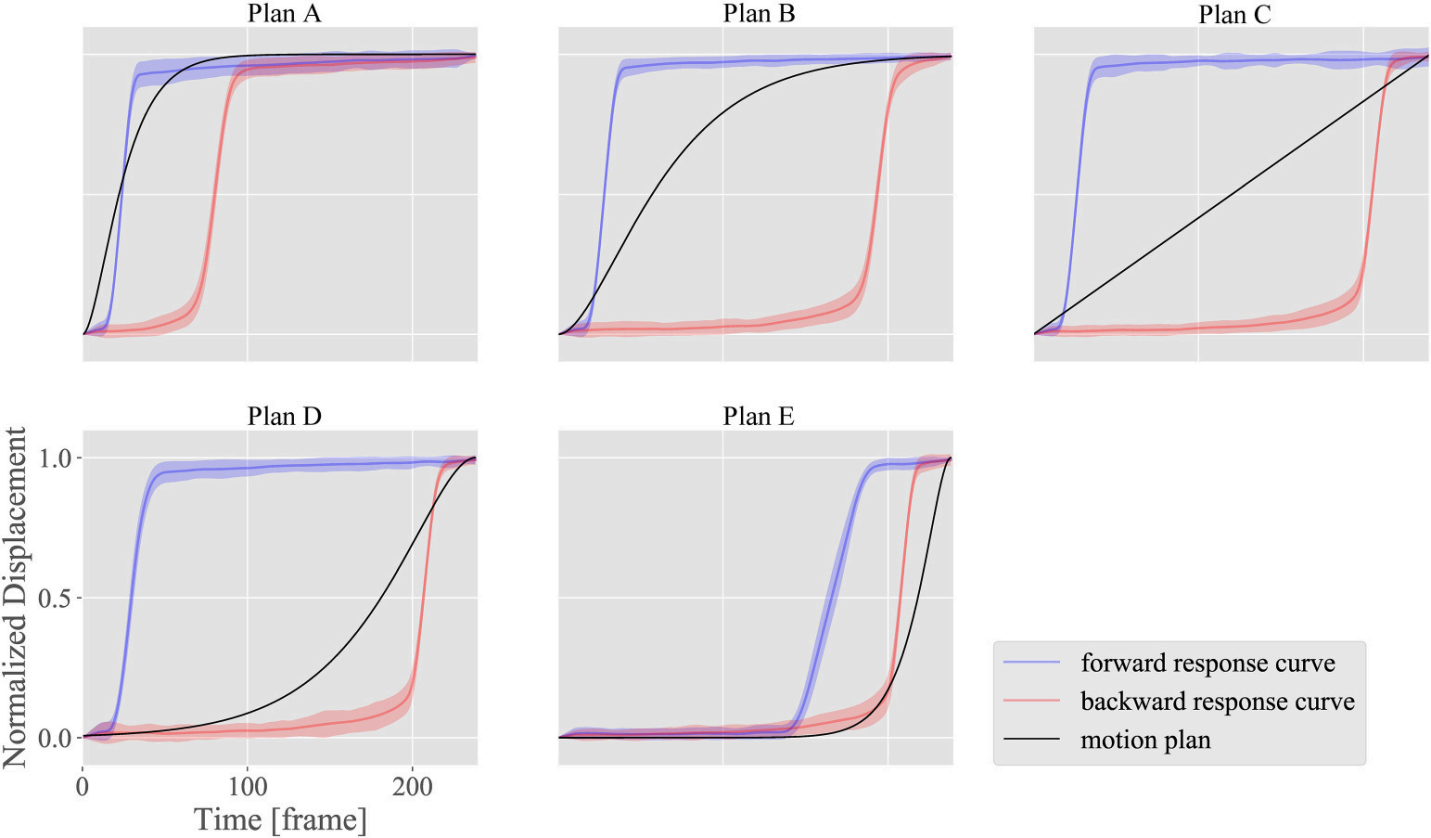
Motion design results for motion plans from A to E with DU 4 (eyebrow raising), which shows large ^*n*^α (sensitivity) and ^*n*^β (hysteresis), and small ^*n*^γ (dyssynchrony).

**Figure 13:**
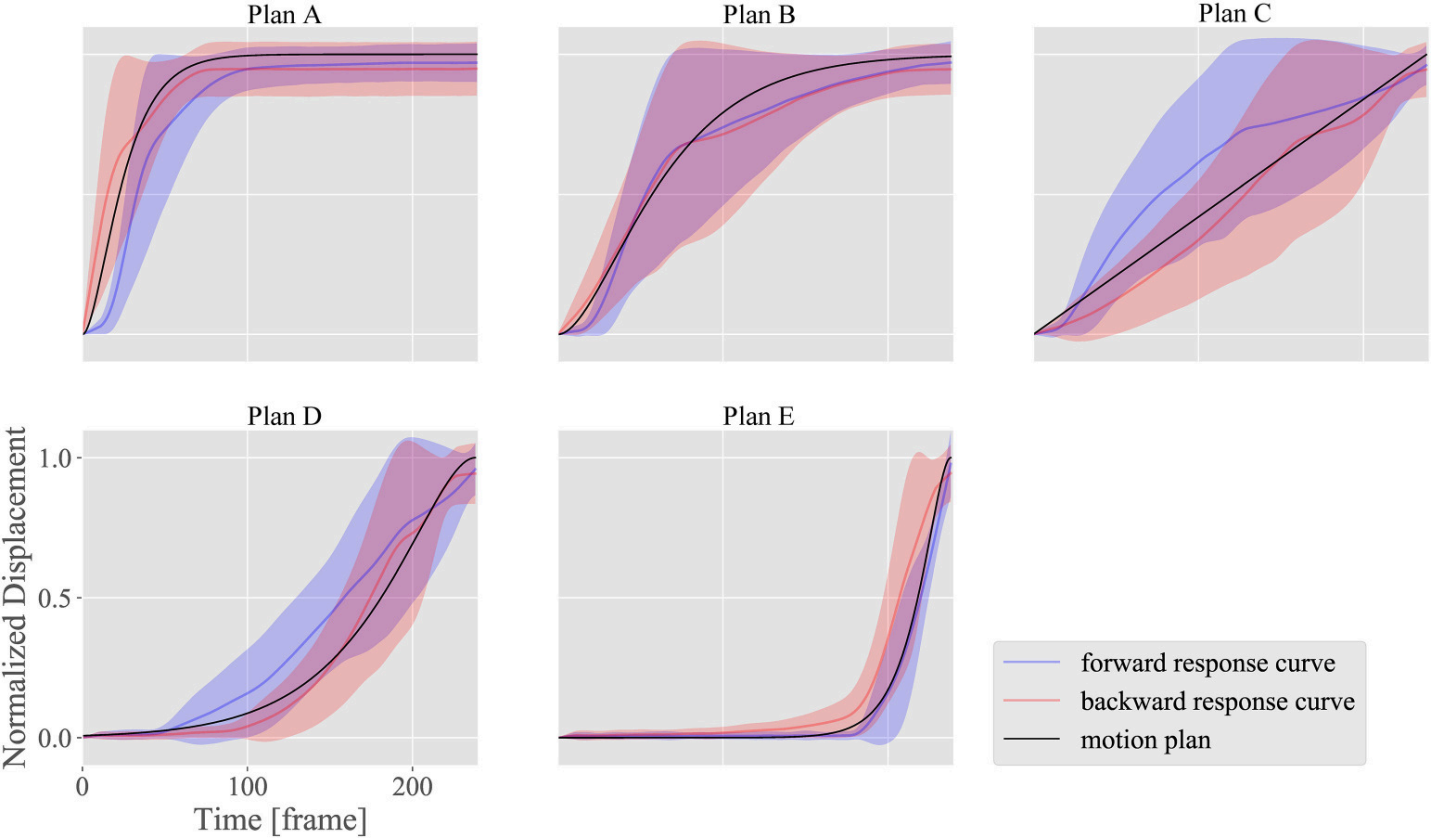
Motion design results for motion plans from A to E with DU 11 (lower lip centering), which shows small ^*n*^α (sensitivity), and ^*n*^β (hysteresis), and large ^*n*^γ (dyssynchrony).

**Figure 14:**
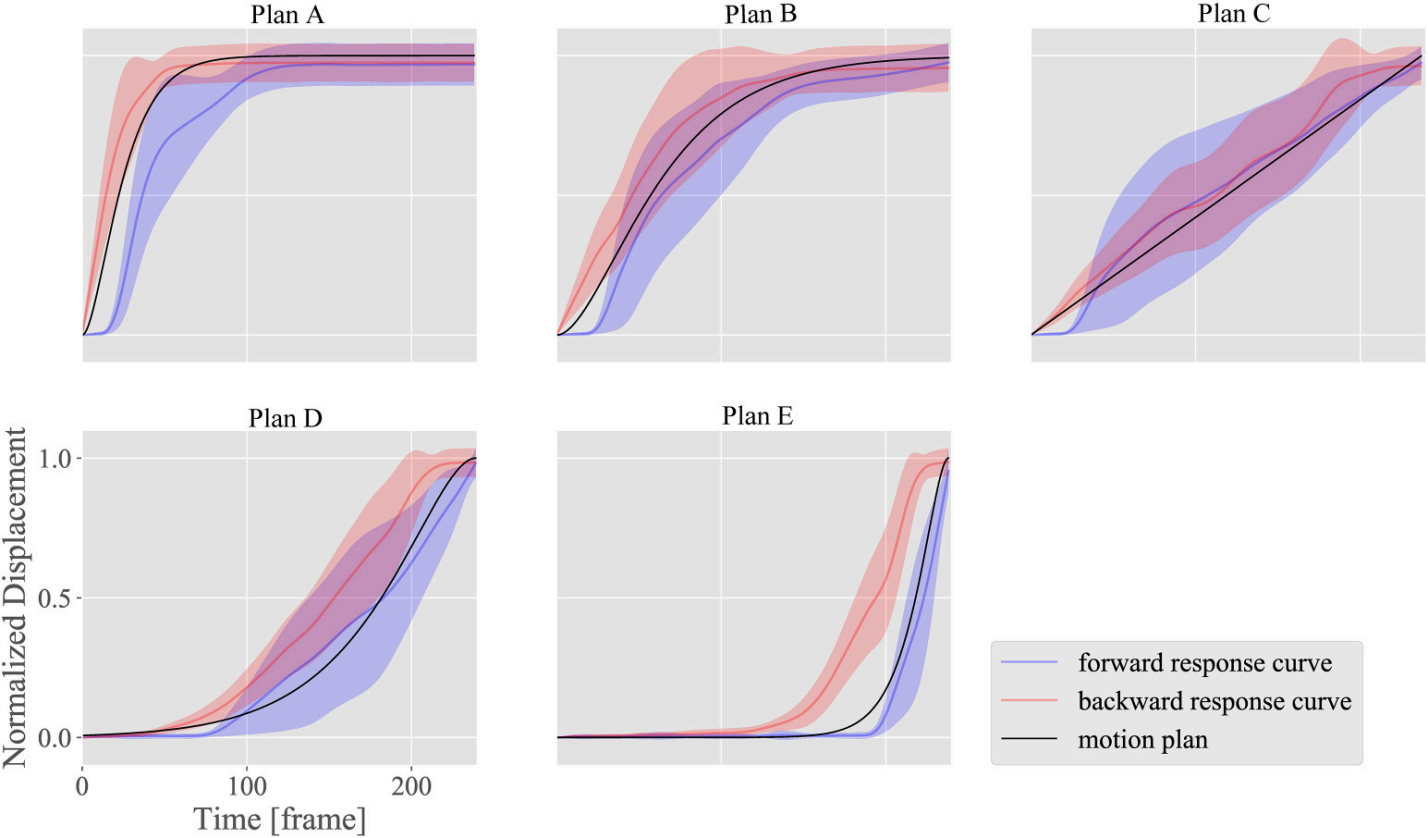
Motion design results for motion plans from A to E applying the combination of DUs 1 (upper eyelid lowering), 7 (middle cheek side-pulling), and 12 (jaw opening), to show a smiling face. Movies of design results for motion plans A, B, and C are available as Supplementary Materials Videos 1, 2, and 3 for this article.

Figures 12–14 show the motion design results for the motion plans from A to E in the three design situations with a DU with large ^*n*^α and ^*n*^β; another DU with large ^*n*^γ; and a combination of DUs 1, 7, and 12, respectively. We used DU 4 (eyebrow raising) and DU 11 (lower lip centering) for the first and second situations, respectively, based on the property chart.

The horizontal axes indicate the measurement time *t*, while the vertical axes indicate the normalized displacement. The black curves represent the plan functions (or ideal motion patterns), and the blue and red curves represent the realized motion patterns in the forward and backward situations, respectively, with standard deviations of the realized motion patterns for all markers.

Figures 12, 13 show how strongly the response properties were reflected in the design results. The realized normalized displacements shown in Figure 12 were increased or decreased largely by small changes in the ideal normalized displacement. Consequently, none of the motion plans were realized with DU 4 (eyebrow raising) because of this large sensitivity. In contrast, the realized normalized displacements shown in Figure 13 averaged very near the ideal ones for every motion plan, although their standard deviations were quite large, indicating that motion patterns induced by DU 11 (lower lip centering) were not homogeneous among markers.

The results also showed that the motion plans implemented with several DUs could be realized with the proposed design methodology. Although the response functions were different among DUs 1 (upper eyelid lowering), 7 (middle cheek side-pulling), and 12 (jaw opening) as shown in Figure 10, the motion patterns realized with the combinations of these DUs approximately followed the ideal motion patterns, indicating that the proposed design methodology canceled the difference in the response curves among these DUs to some degree. However, the realized motion patterns were delayed more than the ideal ones: the former tended to be smaller in forward situations, whereas they tended to be larger in backward situations. This delay was attributed to insufficient hysteresis compensation based on the simple response functions obtained in the limited situations where the commands increase and decrease at the maximum amplitude from 0 to 255.

## 4. Discussion

### 4.1. Complexity of spatial distribution of displacements

Figure 8 indicates that the spatial deformations produced by each DU were generated by displacement vectors with various directions, and the maximum displacements were different among DUs. This complex spatial distribution of displacements suggests that the conventional descriptions of android robot faces, with the number, approximate positions, and representative directions of movable mechanical parts, as shown in Figure 2, is insufficient for precisely representing the system performance because the actual displacement distributions are so complicated that they cannot be estimated from the conventional descriptions.

In addition to the insufficiency of the conventional description method, the results presented here suggest that android face studies are recommended to measure and describe the displacement vector field for each DU as shown in Figure 8. These vector fields can be used to determine which DUs more effectively implement desired deformation patterns and to what extent the deformation areas of several DUs overlap with each other. Moreover, we can compare the vector fields among several android faces to determine quantitatively which faces are more expressive. Furthermore, we can analyze the different deformations between android and human faces, whose displacement vector fields were measured and investigated in our previous study (Ishihara et al., 2017). Analyses of vector field differences between human and android faces will be among our future works.

### 4.2. Face system evaluation based on the property chart

These results also suggest that android faces could be evaluated quantitatively with the property chart shown in Figure 11. This chart shows to what extent the face system is easy to control overall and which DUs and their properties should be improved to enhance overall performance. In the case of Affetto, sensitivity ^*n*^α was larger than 0.03 for almost all DUs without sensory feedback, whereas DUs 1, 2, and 12 with sensory feedback had smaller sensitivities ^*n*^α smaller than 0.03. These results suggested that the sensitivity ^*n*^α for the DUs without sensory feedback could be reduced to approximately 0.03 if sensory feedback could be provided for these DUs.

Hysteresis ^*n*^β was larger than 50 for every DU except DUs 1 and 12. This hysteresis deteriorates the motion design results as shown in Figure 14 and described in section 3.3. There are two promising approaches to avoid the performance deterioration due to the hysteresis: first, following a model-based approach, we conduct additional measurement experiments to obtain more detailed hysteresis models capable of representing to what extent hysteresis occurs when the transition direction of the commands change arbitrarily; second, we fundamentally modify the hardware structure of the face system to reduce the hysteresis elements. The hysteresis becomes larger if the contact conditions between the skin sheet and shell change in a hysteretic manner. Therefore, some improvements to attach the skin sheet on the shell smoothly and elastically are necessary for future android faces.

This property chart is also useful when we quantitatively evaluate to what extent hardware modifications are effective. The modifications can be evaluated well if the property indices become sufficiently small. In addition, the chart is useful for determining which android faces are superior as a mechanical system. Android faces have generally been judged based on vague evaluations of their appearance, and such subjective evaluations are not effective for specifying the problematic facial parts and movements. Thus, the proposed system identification and evaluation method can be used in future android face studies to advance android face system designs.

## 5. Conclusion

This paper presented a design methodology for motion patterns based on system identification of static responses on a child android robot face. Sigmoid functions were used to model the static response curves of its facial surface displacements against increasing or decreasing input commands. The resulting surface motion patterns implemented with single DUs indicated that the proposed static response properties can be used to predict design results. In addition, the surface motion patterns for the neutral to smiling transition implemented with several DUs indicated that the proposed design methodology can cancel the differences among the response curves of each DU. The proposed method can thus be used to identify and evaluate the face system and is an effective solution to the black box problem of android robot faces.

## Author contributions

HI designed the study and wrote the initial draft of the manuscript. BW contributed to data collection and analysis, as well as to the preparation of the manuscript. All authors contributed to interpretation of data and critically reviewed the manuscript.

### Conflict of interest statement

The authors declare that the research was conducted in the absence of any commercial or financial relationships that could be construed as a potential conflict of interest.
